# Assessment of Internal Structure of Spun Concrete Using Image Analysis and Physicochemical Methods

**DOI:** 10.3390/ma13183987

**Published:** 2020-09-09

**Authors:** Jarosław Michałek, Maciej Sobótka

**Affiliations:** Faculty of Civil Engineering, Wroclaw University of Science and Technology, 50-370 Wroclaw, Poland; jaroslaw.michalek@pwr.edu.pl

**Keywords:** concrete centrifugation, morphology, image processing, porosity, aggregate, cement

## Abstract

Taking into account the possibilities offered by two imaging methods, X-ray microcomputed tomography (µCT) and two-dimensional optical scanning, this article discusses the possibility of using these methods to assess the internal structure of spun concrete, particularly its composition after hardening. To demonstrate the performance of the approach based on imaging, laboratory techniques based on physical and chemical methods were used as verification. Comparison of obtained results of applied research methods was carried out on samples of spun concrete, characterized by a layered structure of the annular cross-section. Samples were taken from the power pole E10.5/6c (Strunobet-Migacz, Lewin Brzeski, Poland) made by one of the Polish manufacturers of prestressed concrete E-poles precast in steel molds. The validation shows that optical scanning followed by appropriate image analysis is an effective method for evaluation of the spun concrete internal structure. In addition, such analysis can significantly complement the results of laboratory methods used so far. In a fairly simple way, through the porosity image, it can reveal improperly selected parameters of concrete spinning such as speed and time, and, through the distribution of cement content in the cross-section of the element, it can indicate compliance with the requirement for corrosion durability of spun concrete. The research methodology presented in the paper can be used to improve the production process of poles made of spun concrete; it can be an effective tool for verifying concrete structure.

## 1. Introduction

**Figure 1:**
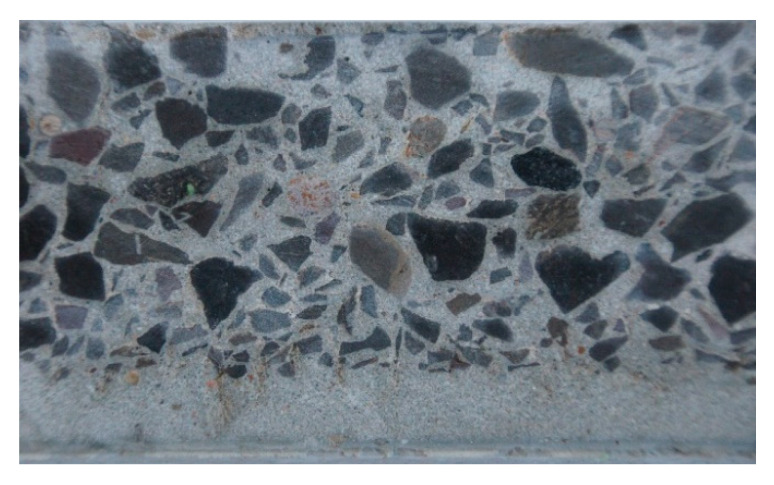
The structure of spun concrete in the wall thickness of a power pole.

Concrete centrifugation is the process of forming and compacting concrete mix due to the normal (radial) force generated during the spinning of the mold around its longitudinal axis at a speed of 500–700 rpm. Due to its specificity, this method is applicable only to hollow elements. As a result of the centrifugation process, concrete with a heterogeneous and layered structure is obtained [1,2,3]. Thus, the structure is different from that of precast or monolithic concrete elements, which in practice can be considered homogeneous. The spun concrete is marked by the fact that components with a larger mass (coarse grains) come to the outside of the cross-section, while components with a smaller mass (cement slurry) tend to remain inside (Figure 1). Under these conditions, the outer layer can achieve high compactness and, after hardening, high strength and resistance to chemical and mechanical impacts. The inner layer, on the other hand, consists of highly compacted, very thick cement paste and may, after hardening, obtain special resistance to water permeability.

One of the first to describe the structure of spun concrete used in pipe production was Marquardt [4]. Based on his observations, he found that a greater difference between the specific gravity of the concrete mixture components led to faster and deeper fractionation. He recommended that well-sorted, mixed aggregate grains with similar petrographic properties and a maximum diameter of 15 mm were preferred for concrete mixtures subjected to centrifugation. He also discussed the change in cement content as a function of the wall thickness of spun concrete elements. He found that cement at 83% of the wall thickness is quite evenly distributed, and an increase in its content is observed only in the inner layer of the section with a thickness of 2–5 mm. He proposed to use layered centrifugation at lower mold rotational speeds to reduce layering.

In [5], Kuranovas and Kvedaras presented the concrete centrifugation process as a four-phase process. The first phase is feeding the concrete mix inside the mold, with its even distribution over the length of the mold at a low centrifuge rotational speed. Afterward, the rotational speed of the mold increases, and the process enters the second phase, where the centrifugal force begins to compress the concrete mix and a layered wall of the concrete section is formed. The third phase is a further increase in the rotational speed of the mold, at which the wall thickness of the concrete section is compacted and stabilized with some of the mixing water squeezed inside. The fourth phase occurs at the maximum rotational speed of the mold, during which the concrete mix is further compacted and excess mixing water is squeezed out. The element’s wall reaches its designed thickness.

Kliukas, Jaras, and Lukoševičienė in [6] found that each phase of the concrete centrifugation process has a specific rotational speed, which depends on the dimensions of the element and the time needed to obtain the selected speed. According to [6], the duration of the first phase should be 3–4 min, the second and third phase should be 1–2 min, and the fourth phase should be 10–15 min. It should be noted that the centrifugation time of an element in a steel form also depends on the initial water–cement ratio of the concrete mix and the pressure caused by centrifugal force, depending on the rotational speed and the diameter of the product.

Adesiyun et al. [7,8,9] used a computer image analysis method to describe the structure of spun concrete allowing extraction of quantitative and qualitative information contained in the image of an element wall cross-section. For a precise description of the structure, the wall thickness of the sample was divided into 20 strips with a width of 2.25–3.00 mm (depending on the overall wall thickness). The tests were carried out with different combinations of concrete mix parameters, such as sand point (25–50%), cement content (410–530 kg/m^3^), amount of plasticizer (0–2%), spinning speed (400–700 rpm), and its time (5–11 min). Computer image analysis allowed for graphical representation of the aggregate, cement matrix, and air-void distribution as a function of the wall thickness in individual samples. It was found that the volume fraction of aggregate decreases from the outside of the centrifuged sample to the inside, while the cement matrix content changes in the opposite direction (in the inner zone, its content is almost 100%). The air content in the cross-section of the wall was higher for internal rather than external layers. Segregation of components was found in all tested samples.

Völgyi et al. [10,11] found that segregation of the concrete mix during centrifugation depends to a large extent on the excess of the cement paste and the degree of compaction. Properties such as strength, porosity, hardness, and composition vary across the wall of the spun concrete due to the segregation of components. The latter can be reduced by using less paste and optimal compaction energy. A relationship between the consistency and the advisable compaction energy was proposed. The formula for the optimal centrifuge settings, i.e., time and speed of spinning, was derived to obtain the best strength properties of the sample.

This paper focuses on methods of assessing the internal structure of spun concrete, which can be used in practice to verify the selection of spinning parameters. In the first part of the work, standard methods for determining the composition of hardened concrete were used to assess its variability across the element wall. Next, based on preliminary results presented in [12], imaging techniques followed by appropriate image analysis were utilized to determine spatial distribution of pores, aggregate, and cement matrix. Comparison of obtained results was carried out for the samples of spun concrete power pole E10.5/6c made by one of the Polish manufacturers of prestressed concrete E-poles. The capabilities and performance of the research methods used were discussed. Conclusions regarding their potential use in practice were formulated.

## 2. Preparation of Test Samples

The E10.5/6c pole used for testing was made in one of the Polish prefabrication plants and was a precast, partly prestressed element shaped as a truncated cone with hollow core. The length of the pole was equal to *L* = 10.5 m. The annular shape of the cross-section resulted from the adopted technology of manufacturing poles by the method of concrete centrifugation in longitudinally unopenable steel mold [13]. The outer diameter of the E10.5/6c column increased from *d_t_* = 173 mm at the top to *d_b_* = 330.5 mm at the base with a constant taper *t* = 15 mm/m. The equivalent characteristic peak resistance force, applied at a distance of 0.17 m from the top, was *P_k_* = 6 kN. The E10.5/6c pole is designed to be produced from C40/50 spun concrete.

When designing power poles made of spun concrete, it was taken into account that they are exposed to direct environmental impacts, described by standard [14], class XC4 (cyclically wet and dry concrete surfaces exposed to contact with water), and for structures located near motorways, class XF2 (vertical concrete surfaces exposed to freezing and de-icing agents from air). The durability of spun prestressed concrete poles is ensured by appropriate thickness of the concrete cover of the reinforcement. Concrete that protects steel against corrosion is marked by limit values describing, among others, the quantity and quality of components (e.g., minimum cement content, water–cement ratio *w*/*c*, and classified aggregates), as well as the minimum compressive strength of concrete and its absorbability. For the XC4 and XF2 environmental exposure class, the standard [14] imposed a minimum cement content of 300 kg/m^3^, maximum *w*/*c* = 0.5, and minimum concrete strength class C30/37. A concrete mix recipe was obtained from the manufacturer for the production of the E10.5/6c pole (Table 1).

**Figure 2:**
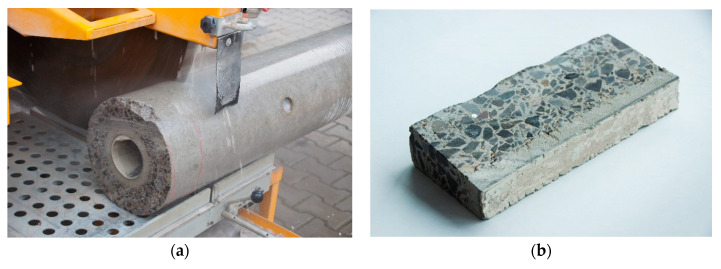
Preparation of test samples: (**a**) cutting off the top of the pole; (**b**) a slice of concrete intended for laboratory testing.

For laboratory tests, from an E10.5/6c power pole, about a 200-mm-long fragment was cut off (Figure 2a) to obtain a sample in the shape of a hollow truncated cone. The wall thickness of the fragment was about 60 mm. The fragment of the pole was then cut along the generatrix into slices about 10–20 mm thick (Figure 2b), with the prestressing wires being avoided. One of the slices was intended for testing the concrete structure with the use of imaging techniques, while the other was intended for testing the composition of hardened concrete using laboratory methods.

## 3. Determining the Composition of Hardened Concrete

For testing the composition of hardened concrete, an estimation method was used in accordance with the instructions [15] based on the determination of apparent density of concrete, the content of parts insoluble in HCl, and the amount of components attached to the binder during its hardening. Since there is no direct method for determining the cement content of a concrete sample, the procedure consists of determining the content of soluble silica and calcium oxide and, on this basis, calculating the cement content. The method is based on the fact that the silicates in Portland cement are much more soluble than the silicate components normally contained in the aggregate. The same applies to the relative solubility of the calcium oxide components in cement and aggregate (except, however, limestone aggregate). The methods for determining the Portland cement content in concrete are described in standards [16,17], as well as in the already mentioned manual [15]. In addition, the extension of the research to determine water absorption and specific density according to [18,19] made it possible to calculate the total and open porosity. The composition of hardened concrete was determined independently for four layers of concrete pole section as shown in Figure 2b.

**Figure 3:**
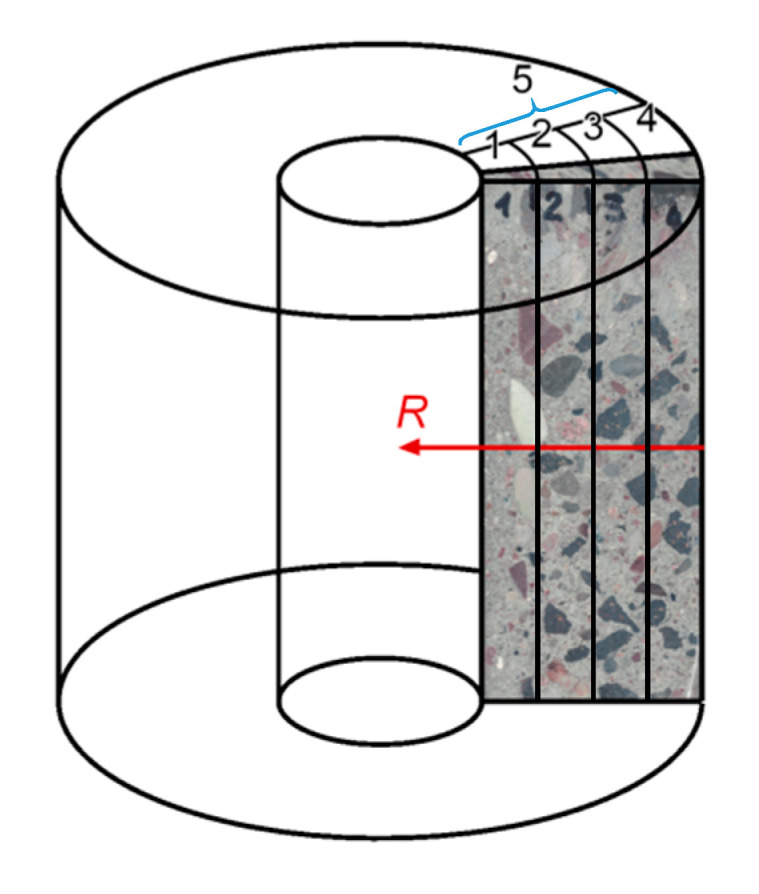
Sample shapes for laboratory tests.

Due to the layered structure of spun concrete, for laboratory tests, samples were prepared by longitudinally cutting the concrete slices (Figure 2b) into four parts (as shown in Figure 3). Samples (layers) were marked with consecutive numbers 1–4, where sample 1 describes the inner layer of the annular cross-section, and sample 4 describes the outer layer. In addition, sample 5 was an entire slice of the concrete wall without splitting into layers. For aggregate with a grain diameter of up to 40 mm, the weight of the concrete sample should be at least 3 kg according to [15]. During the tests, due to the dimensions of separated centrifuged concrete samples, this condition was not met. Moreover, while the concrete slices were being cut, it was not possible to get distinct layers of precise dimensions (as in Figure 1); thus, the boundaries between the layers were in practice inexact, which was especially visible for the inner layer No. 1.

The apparent density of concrete *ρ_c_* was determined via the hydrostatic method due to the irregular shape of the samples. Determination was performed according to [18]. The samples were first dried at 105 °C to constant weight, i.e., until the changes in mass during 24 h were less than 0.2%. Dried samples were weighed after that. Then, the samples were saturated by immersion in water at a temperature of 20 °C until the changes in mass during 24 h were less than 0.2% and weighed. The volume of each sample was determined on the basis of its apparent mass in water and mass in air according to the following formula:

(1) $$V=\frac{m_{a}-m_{w}}{\rho_{w}}$$

where *m_a_* is the mass in air of saturated sample, *m_w_* is the apparent mass in water of saturated sample, and *ρ_w_* = 998 kg/m^3^ is the density of water at 20 °C. When weighing the samples in water, the correction due to apparent mass of suspension wire and holder was made. The apparent density of dry concrete was calculated as

(2) $$\rho_{c}=\frac{m_{o}}{V}$$

where *m_o_* is the mass of dry sample. At the same time, water absorption of concrete can be calculated as

(3) $$n_{w}=\frac{m_{a}-m_{o}}{m_{o}}$$

The procedure for determining the content of parts insoluble in HCl was as follows: after comminution, sieving through a 1-mm sieve, and drying to constant weight at 105 °C, the samples were sieved through a 0.2-mm sieve. Samples of about 2 g were tested using an aqueous solution (1:3) of hydrochloric acid (HCl) to determine the content of insoluble parts. Each sample was placed in a 250-mL beaker and 100 mL of HCl solution was added at room temperature. The contents of the beaker were mixed and the insoluble residue was triturated with a glass rod. After 15 min, the supernatant fluid was decanted. Next, 50 mL of HCl solution was poured onto the precipitate in a beaker and placed in a water bath at 90 °C for 15 min. Then, the contents of the beaker were washed twice with hot water and decanted. The precipitate remaining in the beaker was flooded with 50 mL of 5% Na_2_CO_3_ and placed in a water bath at 90 °C for 15 min, before being washed twice with hot water and decanted. The remaining precipitate was flooded with 50 mL of water, acidified with HCl (1:3) against methyl orange, while adding an excess of 3–4 drops of acid to the neutralized solution, after which the contents of the beaker were filtered. The filtered precipitate was washed six times with hot water until the reaction due to chlorides ceased. The washed precipitate was transferred to a weighed porcelain crucible and, after burning, the filter was calcined at 1000 °C to finally determine the mass of parts insoluble in HCl. Typically, it is assumed that the aggregate mass in the tested sample is equal to the mass of parts insoluble in HCl determined as a result of the analysis. However, when determining the aggregate content in this study, a preliminary analysis was performed to determine the aggregate behavior under the influence of hydrochloric acid. It turned out that basalt in the form of aggregate is resistant to HCl acid, while, after being pulverized, it dissolved partly in hydrochloric acid. Probably weathered basalt was used for concrete in some parts of the aggregate 8–11 mm (Table 1) [20]. Thus, when determining the aggregate content, account was taken of the presence of HCl-soluble parts in the aggregate, by introducing the appropriate correction. Finally, the aggregate mass percentage was calculated as

(4) $$C_{agg,\%}=C_{ins,\%}\cdot corr=\frac{m_{ins}}{m_{o}}\cdot 100\%\cdot corr$$

where *C_ins,_*_%_ is the mass percentage of insoluble parts in concrete, *m_ins_* is the mass of insoluble parts remaining from the sample of concrete, *m_o_* is the initial mass of dry concrete sample, and *corr* = 1/*C_ins,agg,%_* is the correction factor, in which *C_ins,agg,%_* stands for mass percentage of HCl-insoluble parts in the aggregate. The aggregate content in kg/m^3^ was calculated as

(5) $$C_{agg}=C_{agg,\%}\cdot \rho_{c}$$

The content of compounds attached to cement during the setting and hardening of concrete (which are H_2_O and CO_2_) was determined on the basis of calcining losses. The samples were prepared according to the procedure for determining the aggregate content in concrete; they were additionally ground on a 0.06-mm sieve. Calcining losses were determined in an oxidizing atmosphere, while the samples were heated in the air at 950 °C until a constant sample mass was obtained. The constant mass was determined by successive heating for 15 min, followed by cooling and weighing. The percentage calcining loss was determined by the following formula:

(6) $$C_{att,\%}=\frac{\left(m_{o}-m_{cal}\right)}{m_{o}}\cdot 100\%$$

where *m_o_* is the the mass of the sample tested, and *m_cal_* is the the mass of the calcined sample. The obtained value of *C_att_*_,%_ was converted to the content of attached ingredients expressed in kg/m^3^ according to the following formula:

(7) $$C_{att}=\frac{C_{att,\%}}{100\%}\cdot \rho_{c}$$

Finally, the content of cement *C_cem_* was calculated from the following condition:

(8) $$C_{cem}=\rho_{c}-C_{agg}-C_{att}$$

The density of concrete *ρ_cr_*, defined as the ratio of the mass of the dried sample to the volume of the solid part (without taking into account the volume of voids), was determined using the Le Chatelier flask method [19]. On the basis of the values of *ρ_c_* and *ρ_cr_*, total porosity was calculated as

(9) $$\phi_{p,tot}=1-\frac{\rho_{c}}{\rho_{cr}}$$

Open porosity *ϕ_p,op_* was estimated based on water absorption *n_w_* as follows:

(10) $$\phi_{p,op}=n_{w}\frac{\rho_{c}}{\rho_{w}}$$

The obtained parameters of concrete in the particular layers are presented in Table 2.

## 4. Assessment of Morphology of Concrete Microstructure

### 4.1. Theoretical Assumptions

Research on the morphology of the spun concrete microstructure was divided into two parts due to the method of image acquisition: flat optical scanning and three-dimensional (3D) imaging in X-ray microcomputed tomography (µCT). Regardless of the method of obtaining the image, the analysis is aimed at characterizing selected morphology measures of the considered microstructure components: pores, aggregate, and cement matrix. In both cases, the following stages of research can be distinguished:

- obtaining an image;
- segmentation of the component under consideration;
- morphometric analysis.

In the conducted analyses, it was assumed that, due to the axial symmetry of the spun elements, the properties of the obtained material do not depend on the angular coordinate. Thus, at a fixed pole height (i.e., for a fixed coordinate value along the axis of rotation of the element), the parameters of the microstructure change only along the radius. The identified values were, therefore, referred to coordinate *R* in the radial direction (perpendicular to the axis of rotation of the spun concrete element). Coordinate *R* increases from *R* = 0 on the outer edge of the element toward the center of the cross-section in accordance with Figure 3.

The mathematical morphology methods were used to describe the distribution of concrete components (i.e., macropores, cement matrix, and aggregate) over the wall of the spun column. The theoretical foundations for these tools, based mainly on works [21,22,23], were described in more detail in the previous work of the authors [12].

**Figure 4:**
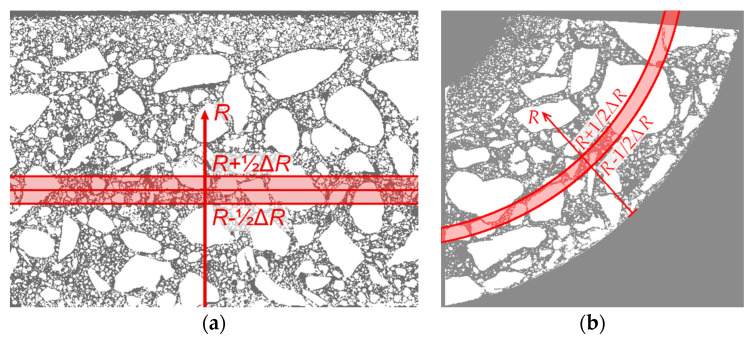
Selection of peripheral band: (**a**) in longitudinal section; (**b**) in cross-section [12].

On the basis of the analyses carried out, selected morphological statistical measures were determined: component volume fraction and so-called local thickness. The average volume fraction of the selected component is defined as the number of pixels in the component divided by the total number of pixels (in a selected area of the image). To describe the variability of the volume of the component as a function of *R*, a series of image subareas was selected, which are narrow circumferential bands of the concrete pole wall. The volume fraction *ϕ*(*R*) (dependent on coordinate *R*) is calculated in the band with the center line corresponding to coordinate *R* and the width Δ*R*. The method of selecting the aforementioned band is shown in Figure 4a. It is worth mentioning that, in the case of cross-section analysis as in [12], the shape of the band is annular (see Figure 4b).

**Figure 5:**
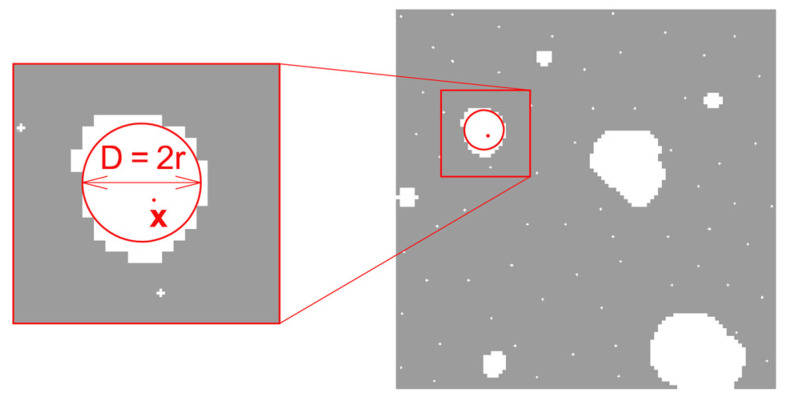
Element thickness at point ***x*** as the maximum diameter of the inscribed circle.

Local structure thickness is a scalar field, defined only in the area occupied by the considered constituent. The procedure for determining the local element thickness generally consists of filling the area occupied by the considered constituent with circles of the largest possible diameter [24]. Then, the thickness of the element at a given point ***x*** is defined as the largest diameter of the circle, which entirely fits within the constituent under consideration and, at the same time, contains point ***x*** in its interior. This is schematically illustrated in Figure 5.

The calculations are performed first for the entire image, resulting in a map of the local thickness of the constituent under consideration. Then, to describe the variation of local thickness as a function of the wall thickness of the concrete element (i.e., relative to coordinate *R*), a procedure analogous to that for the volume fraction was used; specifically, the average in the appropriate circumferential band was determined as in Figure 4.

### 4.2. Two-Dimensional (2D) Imaging in an Optical Scanner

The research procedure used is based on the procedure given in [12]. It consists of the following consecutive actions:

1. cutting the sample;
2. surface preparation for scanning;
3. scanning;
4. segmentation of the considered constituent from the obtained image;
5. morphometric analysis of the considered component based on its binary image.

Staining the pores first and then the matrix (with a different color), after etching it with acid, allowed quantifying the morphology of the following components: air voids (macro-pores), cement matrix, and aggregate. Thus, the scope of analyses was expanded compared to the analysis presented in [12], where only aggregate morphology was described. To perform calculations as a part of the analysis of the morphology of individual components, the author’s own procedure written in the Mathematica program was used. Selected image transformations were carried out in GIMP, ImageJ (Fiji distribution), and CTAn programs.

**Figure 6:**
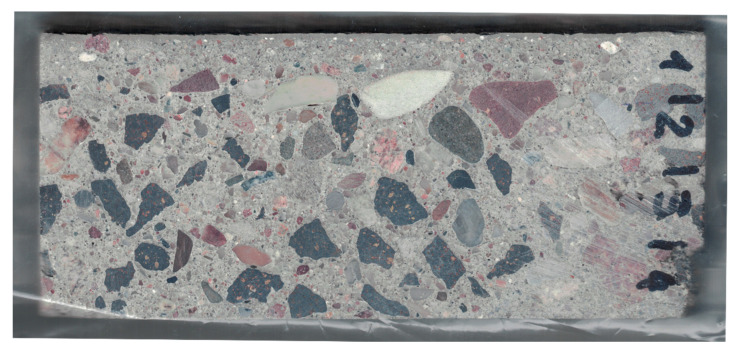
Raw scan of the tested sample.

The first stage of sample preparation for scanning was to level the cut surface in such a way that it was as flat, even, and smooth as possible. For this purpose, a Struers LaboPol 5 grinding and polishing machine with an MD Piano grinding disc was used. The result of the optical scan of the surface of the test sample prepared in this way (before staining) is shown in Figure 6. The scan was performed using a standard office scanner with a resolution of 600 dpi, which, calculated using the pixel size, gives 42.33 µm/pix.

**Figure 7:**
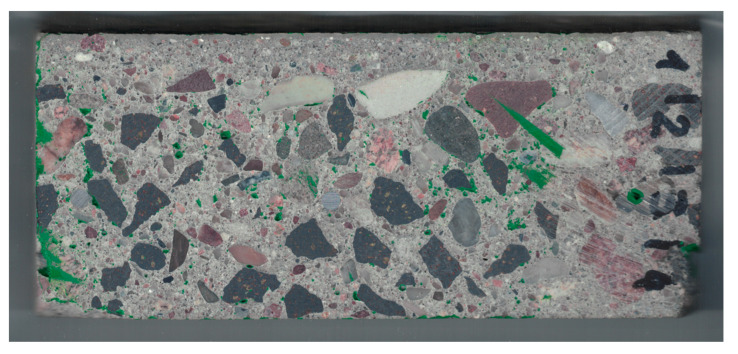
Scan of tested sample after staining the pores.

The pores were stained green by applying acrylic ink to the entire surface of the sample, which was then ground again. After this procedure, the sample was rescanned Figure 7.

**Figure 8:**
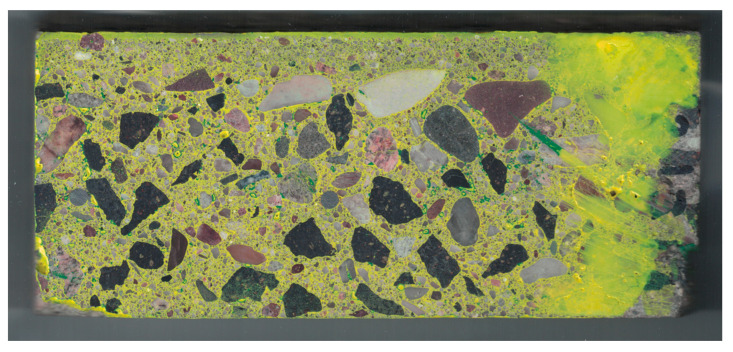
Scan of the tested sample after staining the matrix.

Then, after etching the matrix with hydrochloric acid, it was stained yellow (Figure 8). The method of staining was analogous to that for pores; only the ink color changed.

**Figure 9:**
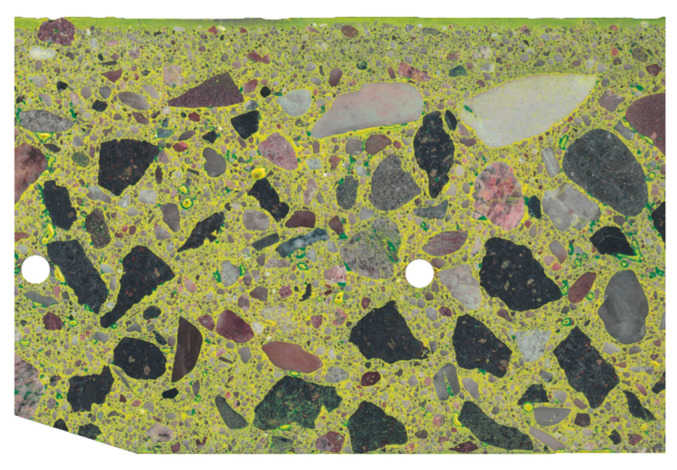
Image of the tested sample after staining the matrix (limited to the region of interest (ROI)).

To enable comparison of scans at individual stages of staining, all images were superimposed and matched to each other by appropriate rotation and shift. The pictures presented below show already “matching” images, for which the dimensions in pixels are the same and the position of the analyzed sample is the same. To exclude the background from the analysis, as well as inadequately polished fragments, edges jagged during cutting, and reinforcing bars, further analysis focused on the area of the image showing the correctly prepared surface of the concrete sample. This area is called the region of interest (ROI). The complement of ROI was not included in the analysis. For this part of the image, a white mask was applied. The image of the stained sample, limited by the aforementioned mask to ROI, is presented in Figure 9. By using ROI, most of the polishing marks, which were stained as pores, were eliminated from the analysis.

#### 4.2.1. Macropore Morphology

**Figure 10:**
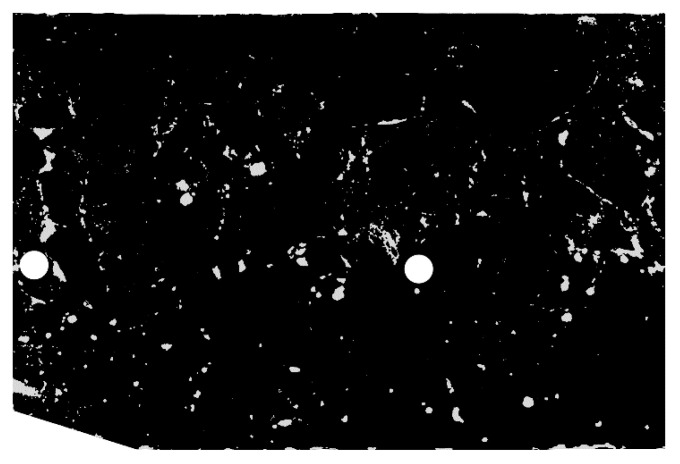
Binary image of pores (limited to ROI).

**Figure 11:**
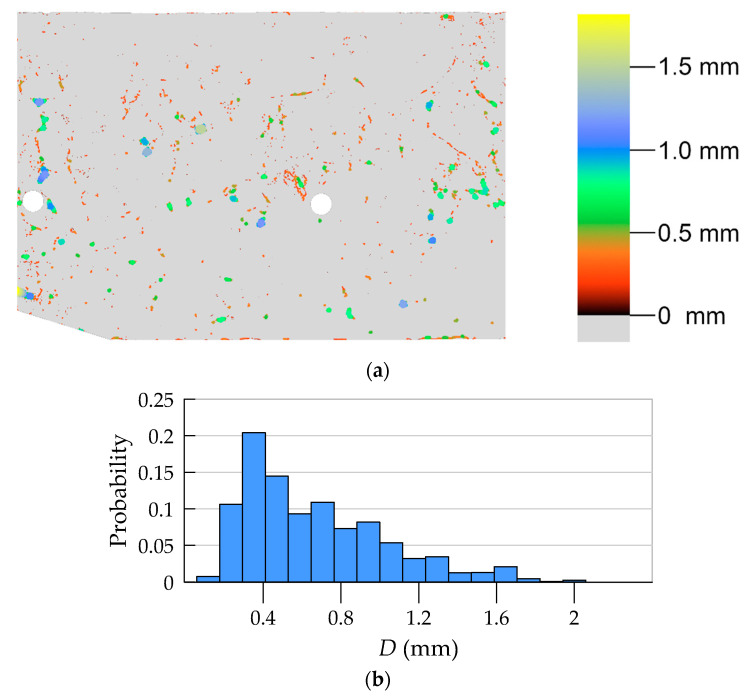
Pore local thickness: (**a**) contour map; (**b**) histogram.

**Figure 12:**
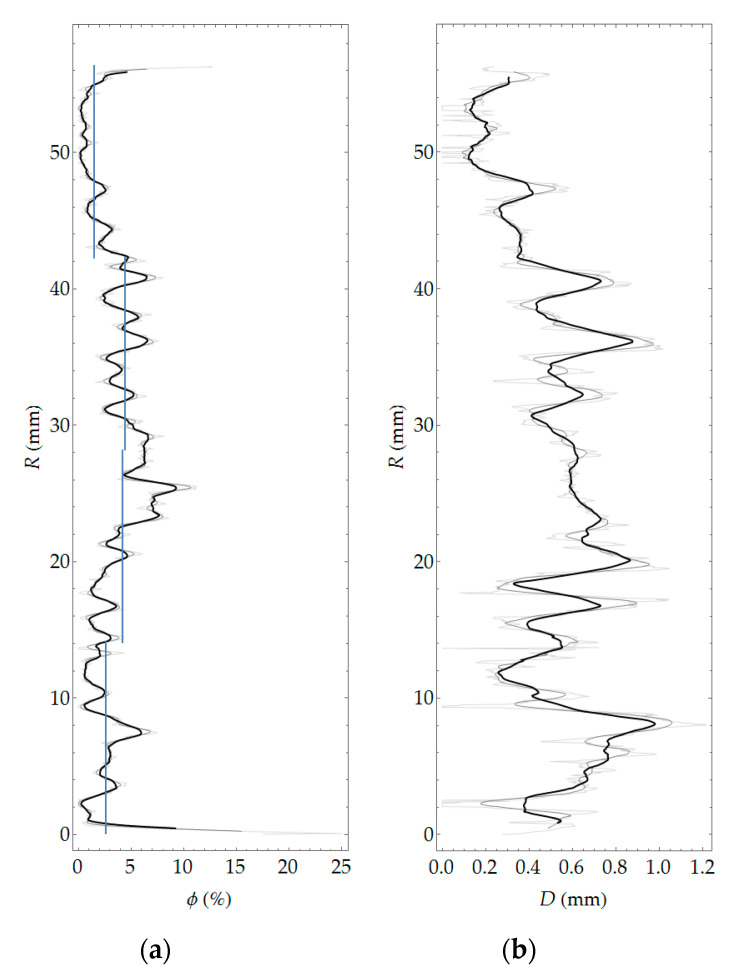
Porosity (**a**) and average local thickness of pores (**b**) as a function of *R* coordinate.

Pore segmentation was performed by subtracting images before and after staining, followed by global thresholding. The particular technique was described in more detail in previous work [12]. The segmentation result, i.e., the binary image of pores, is shown in Figure 10. Figure 11 shows a contour map of pore local thickness, as well as its histogram. Graphs of porosity and average pore thickness as a function of coordinate *R* are presented for the consideration sample in Figure 12. The brightest gray lines correspond to the width Δ*R* (as in Figure 4) equal to the pixel size. Darker lines correspond to greater values of adopted width Δ*R*. The same remark holds true throughout the remaining graphs in the paper.

#### 4.2.2. Cement Matrix Morphology

**Figure 13:**
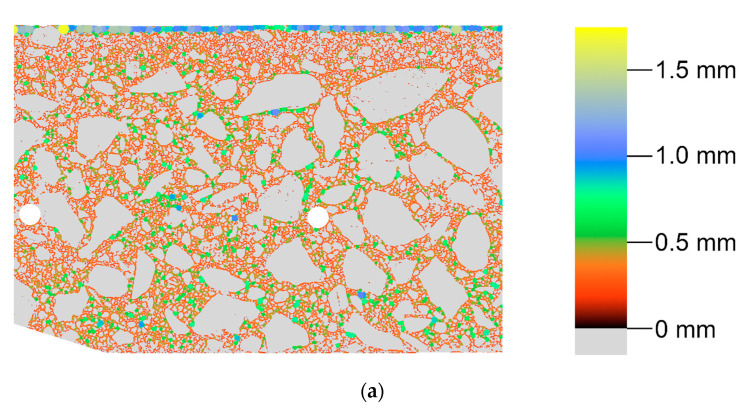

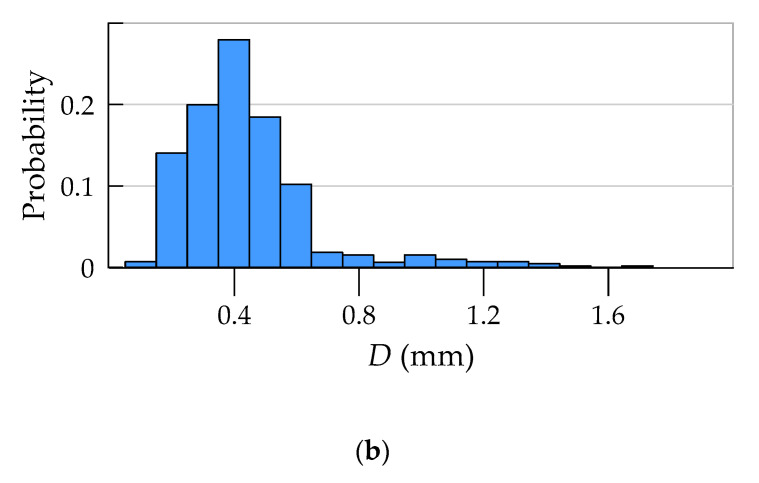
Local thickness of cement matrix: (**a**) contour map (limited to ROI); (**b**) histogram.

**Figure 14:**
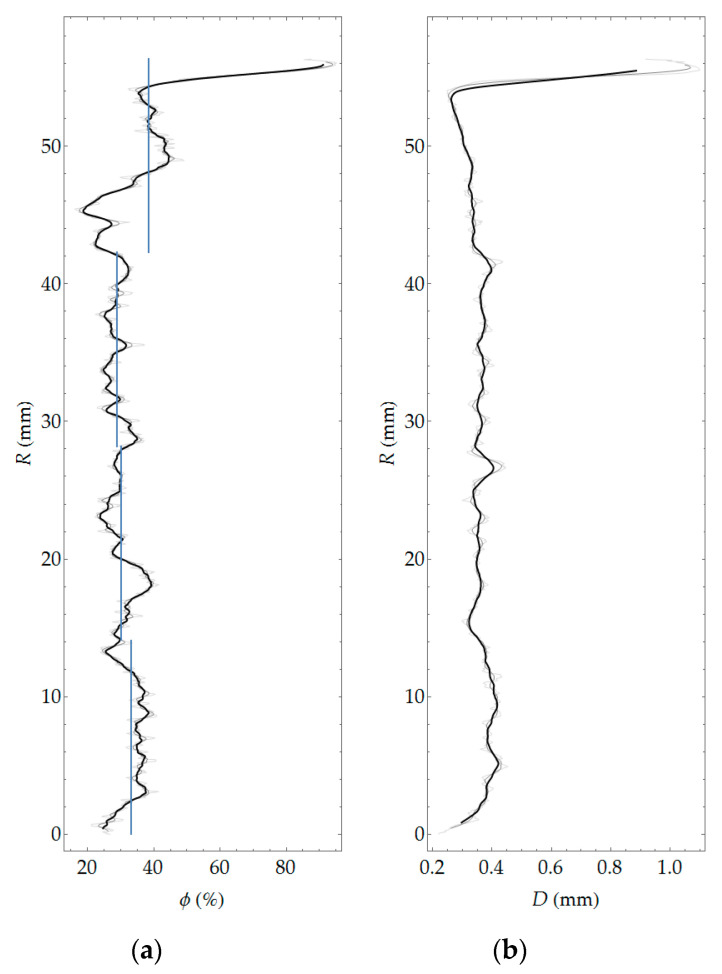
Volume fraction (**a**) and average local thickness (**b**) of cement matrix as a function of the element thickness.

The matrix was segmented by extracting the appropriate color channels from the scan after staining the matrix, followed by thresholding. Next, the pores were removed and the calculations were carried out. Figure 13 presents a map of the local thickness of the cement matrix together with the histogram. Figure 14 shows the variability of the volume fraction and the average local thickness of the matrix as a function of coordinate *R*.

#### 4.2.3. Aggregate Morphology

**Figure 15:**
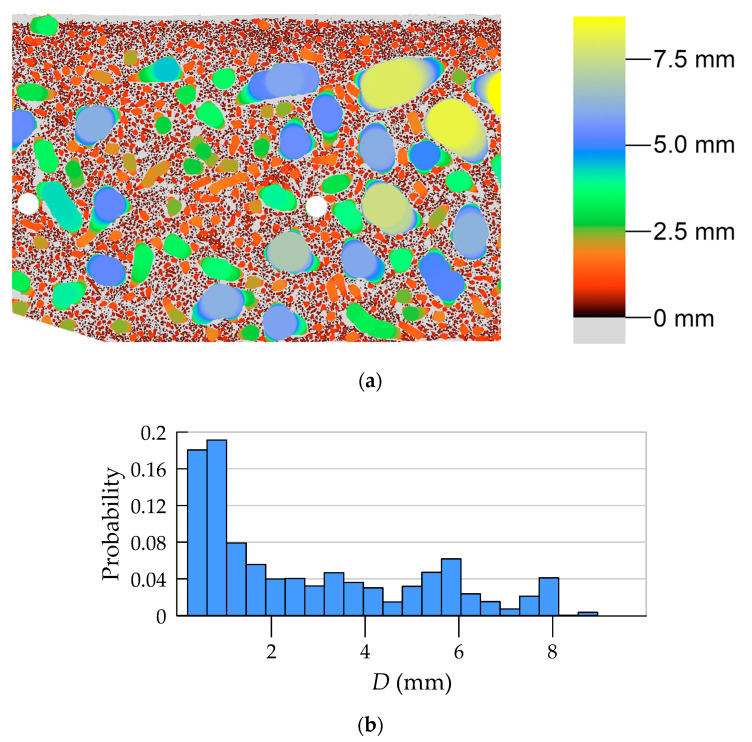
Local thickness of aggregate: (**a**) contour map (limited to ROI); (**b**) histogram.

**Figure 16:**
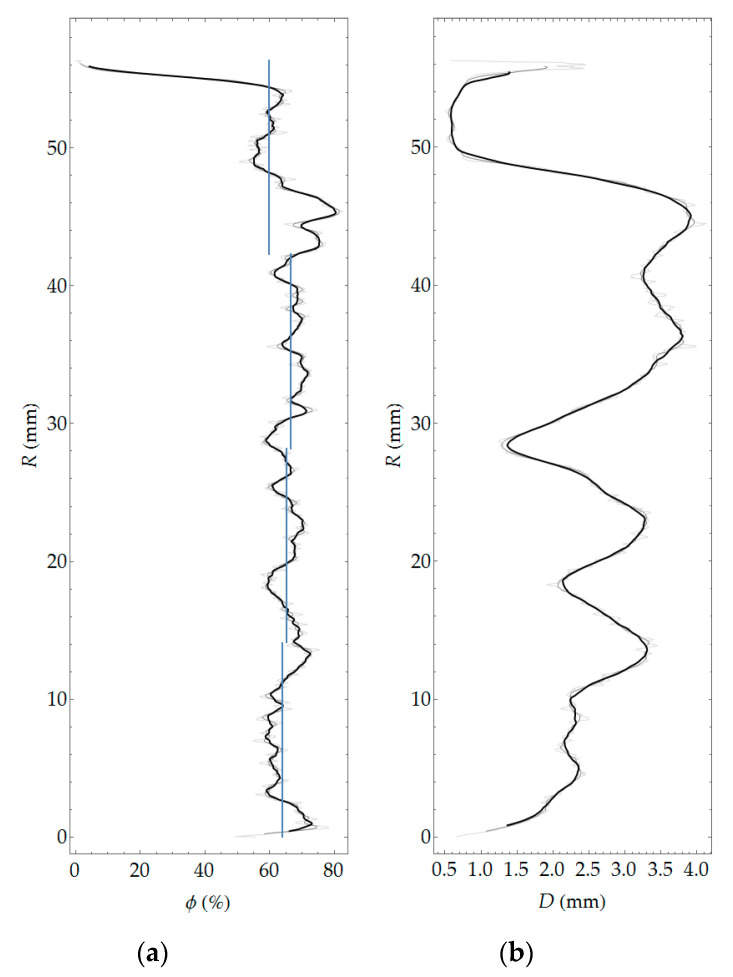
Volume fraction (**a**) and average aggregate thickness (**b**) as a function of coordinate *R*.

The aggregate was segmented as a complement of the area, being the union of the binary images of cement matrix and pores. The map of local aggregate thickness, as well as its histogram, is presented in Figure 15. Figure 16 shows the variability of the volume fraction and average aggregate thickness as a function of coordinate *R*.

### 4.3. 3D Imaging in Microcomputed Tomography

**Figure 17:**
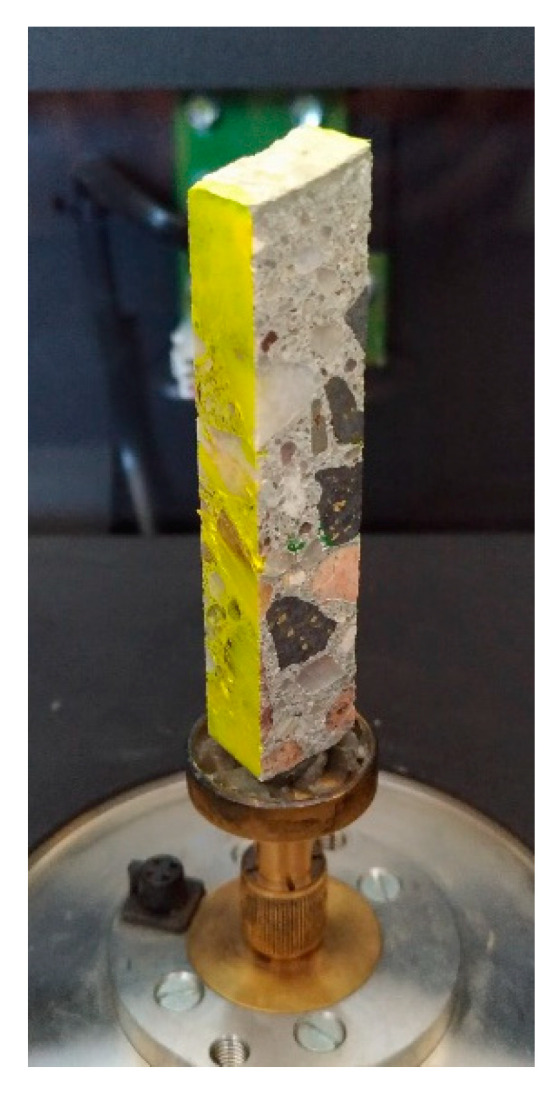
The sample attached to the holder and mounted in the chamber of X-ray scanner.

In order to describe the pore space network, a nondestructive technique, namely, µCT, was used. The sample was cut to a cuboid with dimensions allowing to obtain a test resolution of 21.40 µm/pix. Then, the prepared cuboid was fixed to the holder in the chamber of the device (Figure 17).

Scanning was performed in a Bruker Skyscan 1172 device (Bruker, Kontich, Belgium). The examination consisted of acquiring a series of X-ray projections, followed by the reconstruction of 3D image of the tested sample. The scanning parameters used are shown in Table 3.

Image reconstructions were made using the NRecon program based on the Feldkamp algorithm [25]. The set of reconstruction parameters is presented in Table 4.

**Figure 18:**
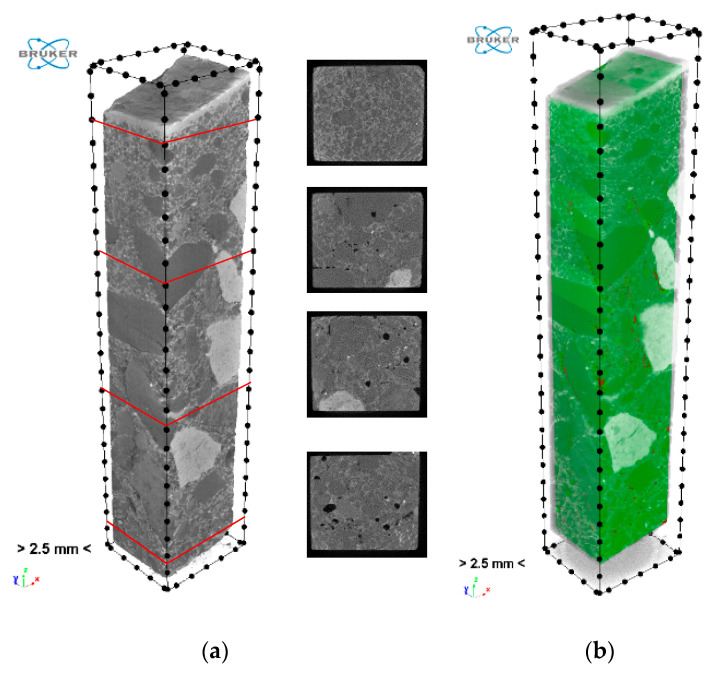
Reconstruction of the tested sample: (**a**) rendered three-dimensional (3D) view with exemplary cross-sections; (**b**) volume of interest (VOI) selection.

The reconstructed structure of the tested sample is shown in Figure 18a. In order to carry out a quantitative and qualitative assessment of the material, it was also necessary to specify in the reconstructed model the volume of interest (VOI). A quasi-cuboidal area was assumed, highlighted in green in Figure 18b.

#### Macropore Morphology

**Figure 19:**
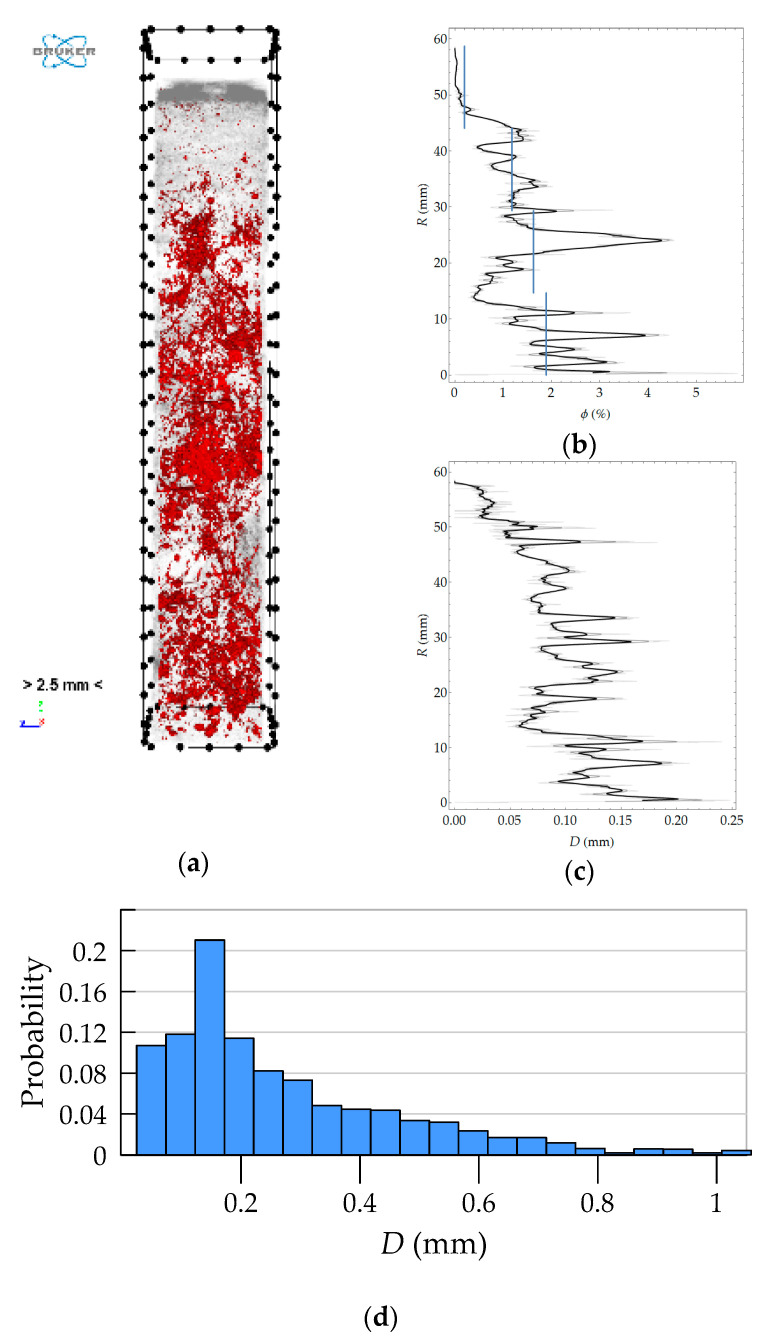
Results of morphometric analysis: (**a**) segmentation of pore space; (**b**) macroporosity as a function of coordinate *R*; (**c**) average local pore thickness as a function of coordinate *R*; (**d**) histogram of pore local thickness.

The first stage of the analysis was pore segmentation using the threshold method preceded by the use of a smoothing filter. The spatial morphology of the pores extracted in this way is shown in the perspective view in Figure 19a, in which the pores are indicated in red and the reconstruction is shown in gray, for which a high level of transparency was set. Morphometric analysis was performed on the binary image of the pore space. In particular, porosity and local pore thickness were determined. The variability of these quantities as a function of coordinate *R* is shown in Figure 19b,c. Figure 19d presents the histogram of pore size. The variation with *R* was determined in a manner analogous to that used in 2D analyses. This time, however, subsequent layers (horizontal sections) of the 3D image were treated as circumferential bands.

## 5. Test Results Obtained Using Different Methods

### 5.1. Pores

In general, all three methods used in the study allow determining porosity. The undoubted advantage of methods based on image analysis is the possibility of obtaining a virtually continuous function of porosity distribution, which in qualitative assessment (whether porosity changes as a function of the element thickness and what is the course of this variability) is very important. In addition, these methods make it possible to evaluate the morphology of pore space, in particular, to determine quantities such as pore width (see Figure 12) or shape parameters, e.g., sphericity. The laboratory method does not boast these advantages; in the field of assessment of porosity distribution, it allows in practice determining only the average porosity values in several arbitrarily determined layers of the element wall. In turn, the main limitation of the methods based on image analysis is linked to resolution. It is possible to obtain information only about pores larger than the pixel size (or voxel in 3D). With the test resolution used, on the order of tens of micrometers, such pores should be defined as structural macropores [26,27]. Obtaining any information about microporosity using methods based on image analysis requires extension of the methodology and the adoption of additional assumptions, e.g., [28,29]. Laboratory tests used in this work allow determining two types of porosity, i.e., total porosity and open porosity. The latter, due to the physical basis of the study (i.e., water absorption of concrete), should be primarily identified with capillary porosity. Apart from the limitations of the research methods used, they should be considered complementary to each other, i.e., giving detailed information on individual types of porosity: structural, total, and capillary (open).

Comparison of the results obtained with all of the methods used is only possible for average values in arbitrarily separated (four) layers of the wall of a spun pole, which corresponds to the division used in laboratory tests. For the results of optical scanning and tomography, the means were calculated on the basis of predetermined porosity distributions as a function of the wall thickness and referred to four layers equal in thickness. The average values of macroporosity in the layers calculated in this way are represented by blue lines in the graphs in Figure 12 and Figure 19. The average values of the porosities in the layers according to all considered research methods are presented in Table 5.

Due to the differences in the research methodology, it is difficult to unequivocally compare the values or mutually verify the results of laboratory and image-based tests, as different types of porosity were determined. Of course, certain constraints should be met, e.g., both macroporosity and microporosity should be less than total porosity. This condition was met.

The comparison of macroporosity results obtained from optical scanning and tomography led to unexpected conclusions. Better resolution used in tomography (smaller pixel size) would suggest the possibility of extracting some portion of small pores, which cannot be observed by the second method with worse resolution. Then, the porosity of the tested sample should be lower in the case of optical scanning, in which the inferior resolution was used [30]. The presented results were the opposite. There can be several reasons for this surprising result. First, the image obtained through tomography results from mathematical reconstruction, and pixel/voxel size should not only be taken as a measure of accuracy. The resolution of the reconstructed image may be impaired due to inaccuracy of the projection images (causing blur) or noise. This may result in a lower porosity value. Secondly, the volume of material, due to the width of the sample subjected to scanning, may be too small to obtain representative results. It is usually assumed in concrete tests that the representative volume element (RVE) dimension should not be less than four aggregate diameters [26]. Fulfilling such a condition while maintaining adequate resolution is in practice very difficult. In the case of optical scanning, on the other hand, the possibility of “tearing out” fine grains when cutting and grinding the surface before scanning should be taken into account. Such an effect could possibly lead to increased porosity.

With regard to the assessment of porosity of the analyzed sample, thanks to the combined use of laboratory methods and imaging, the following findings were obtained:

- capillary porosity (open) is basically constant in all layers and its value is around 10%;
- total porosity is significantly higher only in layer 3 (by approximately 20%) compared to the other layers, where the value of total porosity is between 16% and 17%;
- structural macroporosity (as can be seen from both imaging methods used) shows the largest relative differences between individual layers and is even several times smaller in the middle part (i.e., in layer 1) compared to the other layers.

### 5.2. Aggregate

The aggregate content was determined using two methods: in laboratory tests and on the basis of plain optical scanning. General comments on the possibilities of the research techniques used are very similar to those given in Section 5.1. The surface scanning combined with image analysis gives the possibility of obtaining a virtually continuous function of the aggregate content distribution, as well as other morphology measures, characterizing, e.g., grain size (Figure 15 and Figure 16). The laboratory method allowed determining the average values of aggregate content in four layers of the element wall. As in the case of pore analysis, the results of optical scanning had to be averaged within the four layers of equal thickness. Averaged values are presented in blue lines in the graph in Figure 16. In addition, to enable comparison of results from both methods, the volume fraction of aggregate was converted into its content as follows:

(11) $$C_{agg}=\rho_{agg}\phi_{agg}$$

where *C_agg_* is the aggregate content (kg/m^3^), *ϕ_agg_* is the volume of aggregate, and *ρ_agg_* is the specific density of aggregate.

To apply Equation (11), it is necessary to know the specific density of aggregate *ρ_agg_* in individual layers. Unfortunately, this value was not explicitly determined as part of the research. Therefore, two approaches were taken to estimate this value. In the first approach (*), a constant value equal to the average density of the aggregate used for the mixture was assumed, i.e., *ρ_agg_* = 2710 kg/m^3^; thus, its segregation (variability in wall thickness) due to centrifugation was not taken into account. In the second approach (**), it was assumed that the specific density of all solid particles is a sufficiently accurate estimation of aggregate density, i.e., *ρ_agg_* ≈ *ρ_cr_*. This approach takes into account the variability due to centrifugation, and the estimation error should not be large, because aggregate represents the vast majority of the weight of the solids in the concrete mix (almost 80%); moreover, cement density is similar to aggregate density. Finally, regardless of the adopted estimation, good compliance of laboratory test results and imaging was obtained (Table 6). Relative differences in the results obtained using different methods did not exceed a few percent, while the values in individual layers differed by up to 20%.

While analyzing the results in Table 6, it should be borne in mind that, in the case of imaging, only the aggregate volume fraction *ϕ_agg_* was determined intrinsically. Calculation of mass content in this case required additional data, which could be determined from the recipe for the mixture and physical characteristics of its components, or even on the basis of laboratory test results. However, the obtained compliance indicates that the results of both methods are consistent; the aggregate morphology determined from scanning corresponds to the average values of its content from tests based on physicochemical methods. The results of both test methods used can be considered complementary; they can be used together to analyze spun concrete structure. In particular, thanks to the use of known relationships (e.g., Equation (11)), by creating an appropriate correlation, the image analysis can be used to “extend” point information (in layers) to a continuous functional distribution as a function of the wall thickness.

The content of coarse aggregate decreases toward the inner cross-sectional area. The last inner layer is marked by almost zero aggregate content (Figure 16). It is interesting that the aggregate content as a function of the wall thickness is constant in all layers (Figure 16 and Table 6) except for the very inner layer (layer 1). The results of aggregate distribution as a function of the wall thickness of the cross-section confirm the authors’ expectations and macroscopic observations, and they are consistent with the literature [9]. The aggregate content determined using both methods approximately corresponds to the amount of aggregate declared in the mixture recipe given by the manufacturer of spun concrete poles (Table 1).

### 5.3. Cement Content

From the optical scan, the volume fraction of the cement matrix can be determined. Similarly to the analysis of other concrete components, it is possible to assess the variability of the matrix volume fraction as a function of the wall thickness (see Figure 14). The laboratory test determines the average cement content expressed as the mass of cement used per volume unit of hardened concrete. To determine the relationship between these quantities, both of which in fact show the amount of cement, it is necessary to use some additional information. In order to compare the results obtained using different techniques, the content *C_cem_* (kg/m^3^) was referred to, because the requirements for the minimum value of this parameter are provided by the standard [14]. Cement content can be defined as

(12) $$C_{cem}=\phi_{mat}\cdot \left(1-\phi_{\mu p,mat}\right)\cdot C*$$

where *ϕ_mat_* is the volume share of cement matrix (including capillary micropores) according to the estimation made on the basis of optical scanning, *ϕ_µp_,_mat_* is the cement matrix microporosity, and C* is the mass of cement used per volume unit of solid parts of hardened cement matrix (including nonevaporable gel water); according to the relationships given in [31], C* = 1473 kg/m^3^ was assumed.

In Table 7, a comparison of obtained results is given. Due to the lack of detailed information on the microporosity of the matrix, an upper and lower estimation was made, assuming the following as the limit assumptions: (1) an even distribution of porosity within the matrix and aggregate and (2) full tightness of the aggregate, i.e., that all micropores are located in the cement matrix. The microporosity of the matrix corresponding to such assumptions *ϕ_µp,mat_* can be estimated from the following inequalities:

(13) $$\frac{\phi_{p,tot}-\phi_{p,m}}{\phi_{mat}+\phi_{agg}}\leq \phi_{\mu p,mat}\leq \frac{\phi_{p,tot}-\phi_{p,m}}{\phi_{mat}}$$

where *ϕ_p,tot_* is the total porosity (from the laboratory test), *ϕ_p,m_* is the macroporosity (based on scanning, average value in the layer), *ϕ_mat_* is the volume fraction of cement matrix, and *ϕ_agg_* is the volume fraction of the aggregate.

**Figure 20:**
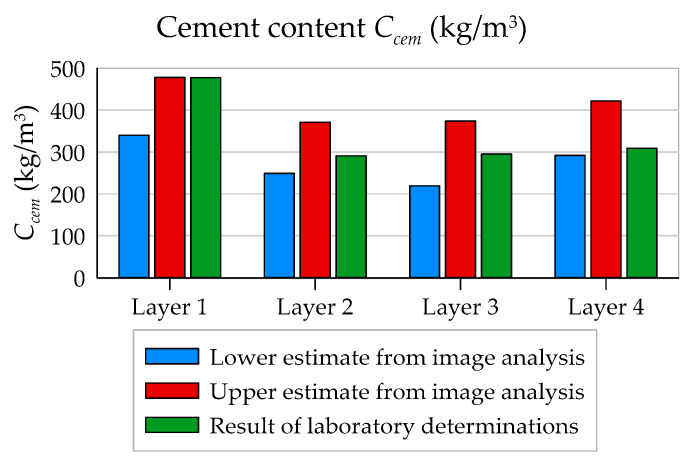
Estimation of cement content *C_cem_* (kg/m^3^) in particular layers of the element’s wall.

The specified ranges in Table 7 are quite wide; however, in terms of quality, cement content is “well rendered”, i.e., the smallest values are in the middle layers (No. 2 and 3), and the highest value is in the inner one (Layer 1) (see Figure 20).

The conclusion concerning the applied research methodology is similar to the previous analyses. The results are comparable and consistent; they complement each other. Imaging naturally results in geometrical relationships, i.e., a description of the cement matrix morphology, which is important information from the point of view of determining the effective parameters of concrete as composite using micromechanics tools or the theory of homogenization [21,32,33,34]. Information on the structure of concrete obtained from laboratory methods is limited to average values of cement content in arbitrarily separated layers. This information directly refers to the practical aspects of design and construction, related, for example, to the formulation of a recipe and control of the composition of concrete mix.

The description of the cement distribution as a function of the wall thickness of the cross-section obtained by means of both methods allows for straightforward qualitative description of this distribution. Optical scanning allowed determining the volume fraction of the cement matrix (Figure 12), with the cement matrix being segmented as a homogeneous component, although in fact it may contain a certain amount of the finest aggregate fraction. However, the laboratory study determined the average cement content expressed as the mass of cement used per volumetric unit of hardened concrete (Table 2). To determine the relationship between these quantities, some additional information was necessary. Due to the lack of detailed data on the microporosity of individual components (aggregate and matrix), a final estimation on the basis of the method of image analysis was made for the lower and upper cement content bounds in individual layers of the element wall (Table 7 and Figure 20). The obtained results show that the cement distribution was relatively constant as a function of the wall thickness, and an increase in its quantity was observed only in the inner layer of the cross-section (2 mm thick) (Figure 14 and Table 7). These research results coincide almost exactly with Marquardt’s conclusions [4].

Cement content in individual layers determined by the laboratory method (Table 2) and by the image analysis method (lower estimate, Table 7) was lower than the amount of cement declared in the recipe by the manufacturer of spun concrete poles (Table 1). It can also be stated that, in the outer layer, the amount of cement, as a result of the centrifugation process, was reduced from 400 kg/m^3^ (Table 1) to about 308 kg/m^3^ (according to the laboratory method, Table 2) and to about 292 kg/m^3^ (according to the lower estimation from the image analysis method, Table 7). Due to the durability of power poles made of spun concrete exposed to direct influence of atmospheric factors, described by standard [14] class XC4 (cyclically wet and dry concrete surfaces exposed to contact with water), and for structures located near motorways class XF2 (vertical concrete surfaces exposed to freezing and de-icing agents), the minimum cement content in the outer layer should not be less than 300 kg/m^3^.

### 5.4. General Remarks

**Figure 21:**
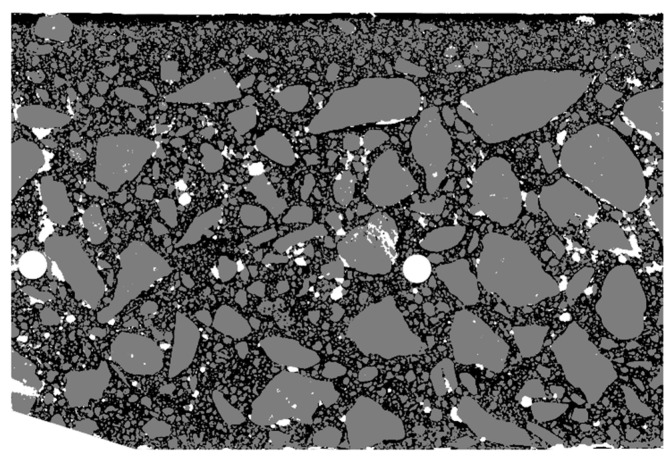
Arrangement of pores (white), aggregate (gray), and cement matrix (black) in the section of the element wall.

It was expected that, during concrete centrifugation, air and water would be squeezed out more from the outer, more compressed layers. Approaching the inner surface of the concrete section, less water would be squeezed out. From the inner layer of the concrete section, where the radial pressure is close to zero, water would not be removed at all. Following laboratory tests, it was observed that the total porosity of the inner layer (No. 3) increases (Table 2) compared to the porosity of other layers. This result confirmed the authors’ expectations and previous research [5,7,8,9], in which it was found that the total porosity of concrete is greater for the inner layers than the outer layers. The other observation made in [7,8,9] was confirmed using image analysis methods. It was observed (Figure 21) that the pores (mostly in layer No. 3) were usually located next to the edges of large grains of aggregate on its outer side. Furthermore, the cement matrix had distinctly fewer aggregate inclusions in these locations compared to the other ones. Therefore, water and air were blocked there from being moved toward the inside of the cross-section as imposed by the centrifugal forces. Such a picture of the porosity and fine aggregate arrangement next to large aggregate grains indicates that the speed and spinning time of concrete in the pole were probably incorrectly selected, preventing the escape of excess air and water from the concrete mixture.

## 6. Summary and Conclusions

The aim of the article was to compare different methods for determining porosity, as well as cement and aggregate content, in the layered structure of spun concrete. The variability of these parameters considered as a function of the thickness of the concrete wall determines the macroscopic properties such as durability, load capacity, and rigidity of spun concrete poles. The article compares the results obtained using two methodologically different approaches: laboratory determinations based on chemical and/or physical methods and image analysis. In general, mass content per unit volume is obtained in laboratory tests, and the volume fraction of individual concrete components is obtained using image analysis methods. If the specific density of the component under consideration is known, conversion of one quantity to another becomes easy.

The main advantage of the methods based on image analysis is the possibility of obtaining a practically continuous function of the content of the considered concrete components as a function of the wall thickness of a spun pole. In this context, the main limitation of chemical/physical methods is the ability to determine only the average value for a few, arbitrarily separated layers of the concrete wall of a spun pole. In turn, the main limitation of image analysis methods is the pixel/voxel size and image quality, particularly due to sample preparation and image acquisition method.

In terms of concrete porosity, it should be stated that the attempt to validate the results obtained from imaging the structure of concrete using laboratory techniques (determining the composition of hardened concrete on the basis of physical and chemical methods) was only partially successful. The results obtained cannot be directly compared due to the fact that porosities are described on different scales. The methods based on image analysis allowed describing the structural porosity (i.e., macroporosity) of concrete, while the laboratory methods enabled describing total and open porosity (capillary microporosity). Quite importantly, imaging methods give the opportunity to analyze the macropore morphology. In particular, it was possible to visualize the location of air voids in relation to the other concrete components (Figure 21), which may have a direct impact on the assessment of the centrifugation process in terms of centrifugation parameters selection such as speed and time.

In terms of the description of aggregate distribution as a function of the wall thickness of the cross-section, very good compatibility of both methods was obtained. The image analysis method allowed determining the volume fraction of aggregate *ϕ_agg_*. Calculation of aggregate mass content per unit volume in this case required additional information about the density of the aggregate, which could be determined either from the recipe for the concrete mixture and the physical characteristics of its components or from laboratory tests. The obtained consistency of the results from both methods shows that the methods are consistent, and the aggregate morphology determined by scanning corresponds to the average values of its content coming from research based on physicochemical methods.

Very good qualitative and quantitative compatibility of both methods in the field of aggregate morphology as a function of the wall thickness of the element was obtained. Thanks to the positive validation of laboratory methods and the method based on image analysis, it can be safely stated that optical scanning is a cheap, relatively fast, and effective method of assessing aggregate segregation as a function of the wall thickness of a spun concrete element.

The description of cement distribution as a function of the wall thickness of the element cross-section obtained using both methods allows for straightforward qualitative description of this distribution. However, full validation success was not achieved because the results based on optical scanning allow obtaining quantitative results only after taking into account additional calculations and information from laboratory tests. Thus, in this case, to get full information about the distribution of cement as a function of the wall thickness of the element, both methods must complement each other. Nevertheless, the information obtained as a result of testing with any of methods used allows assessing the cement content, which determines the requirement for durability of columns made of spun concrete.

An attempt to validate the imaging method with the use of laboratory techniques based on physical and chemical methods showed that optical scanning methods are relatively effective, and they can be a significant complement to the research methods used so far. The analyses also showed that there is a further need to conduct research in the area of spun concrete structure, with particular emphasis on the distribution of concrete porosity and cement content as a function of the wall thickness of the ring-shaped cross-section. The research methods presented in the work can be used to improve the production process of poles made of spun concrete as an effective tool for testing its structure.

## Figures and Tables

**Table 1:** Recipe for concrete mix used in the production of E10.5/6c pole.

Component	Quantity (kg/m^3^)
Sand 0–2 mm	750
Gravel 2–8 mm	350
Gravel 8–16 mm	400
Basalt grit 8–11	370
CEM I 42.5R cement	400
Plasticizer	0.5
Superplasticizer	1.6
Water	105
*w*/*c* ratio	0.26

**Table 2:** Test results and calculations for individual samples 1–4 and average sample 5 (Figure 1).

Parameter	Sample No. 1	Sample No. 2	Sample No. 3	Sample No. 4	Sample No. 5
Apparent density *ρ_c_* (kg/m^3^)	2086	2170	2124	2175	2149
Specific density *ρ_cr_* (kg/m^3^)	2509	2597	2640	2592	2599
Water absorption *n_w_* (%)	5.1	4.4	4.8	4.5	4.6
Open porosity *ϕ_p,op_* (%)	10.7	9.5	10.3	9.7	10.0
Total porosity *ϕ_p,tot_* (%)	16.9	16.4	19.6	16.1	17.3
Solid volume fraction *s* (%)	83.1	83.6	80.4	83.9	82.7
Aggregate mass percentage *C_agg,_*_%_ (%)	71.6	82.5	82.3	81.2	78.2
Aggregate content *C_agg_* (kg/m^3^)	1493.9	1790.6	1748.0	1766.4	1680.5
Attached ingredients mass percentage *C_att,_*_%_ (%)	5.5	4.1	3.8	4.6	3.7
Content of attached ingredients *C_att_* (kg/m^3^)	114.8	89.0	80.7	100.1	79.5
Cement mass percentage *C_cem,_*_%_ (%)	22.9	13.4	13.9	14.2	18.2
Cement content *C_cem_* (kg/m^3^)	477.8	290.8	295.2	308.9	391.1

**Table 3:** Selected, more important scanning parameters.

Parameter	Value
Source voltage (kV)	100
Source current (µA)	100
Image pixel size (µm)	21.38
Filter	Al 0.5 mm
Exposure (ms)	270
Rotation step (°)	0.32
Frame averaging	On (8)
Random movement	On (10)
Use 360 rotation	Yes
Geometrical correction	On

**Table 4:** Selected, more important reconstruction parameters.

Parameter	Value
Pixel size (µm)	21.39861
Object bigger than field of view (FOV)	Off
Ring artefact correction	9
Beam hardening correction (%)	30
Threshold for defect pixel mask (%)	0
CS static rotation (°)	−49.14
Minimum for CS to image conversion	0.000
Maximum for CS to image conversion	0.070

**Table 5:** Comparison of results for different types of porosity.

Parameter	Layer 1	Layer 2	Layer 3	Layer 4
Total porosity from laboratory test *ϕ_p,tot_* (%)	16.9	16.4	19.6	16.1
Capillary porosity (open) from laboratory test *ϕ_p,op_* (%)	10.7	9.5	10.3	9.7
Macroporosity based on optical scanning *ϕ_p,m_* (%)	1.5	4.5	4.3	2.7
Macroporosity from tomography *ϕ_p,m_* (%)	0.2	1.2	1.6	1.9

**Table 6:** Comparison of results (aggregate content).

Parameter	Layer 1	Layer 2	Layer 3	Layer 4
Aggregate content based on laboratory determinations *C_agg_* (kg/m^3^)	1494	1791	1748	1766
Aggregate content calculated using optical scan results (*) *C_agg_* (kg/m^3^)	1505	1732	1729	1661
Aggregate content calculated using optical scan results (**) *C_agg_* (kg/m^3^)	1620	1801	1769	1731

**Table 7:** Comparison of results (cement content).

Parameter	Layer 1	Layer 2	Layer 3	Layer 4
Cement content based on laboratory determinations *C_cem_* (kg/m^3^)	477.8	290.8	295.2	308.9
Upper cement content estimation calculated using optical scanning results *C_cem,max_* (kg/m^3^)	478	371	374	422
Lower cement content estimate calculated using optical scanning results *C_cem,min_* (kg/m^3^)	340	249	219	292

## References

[1] Michałek J. (2016). Concrete lighting poles formerly and nowadays. Mater. Bud..

[2] Michałek J. (2002). Properties of spun concrete in the durability aspect of structures. Durability of Buildings and Protection Against Corrosion, Proceedings of the 13th Conference Kontra 2002, Zakopane, Poland, 22–25 May 2002.

[3] Trapko T., Michałek J. (2012). Using of composite materials for strengthening of spun concrete electrical poles. Przegląd Elektrotechniczny.

[4] Marquardt E. (1930). Geschleuderte Beton- und Eisenbetonrohre. Die Bautech..

[5] Kuranovas A., Kvedaras A.K. (2007). Centrifugally manufactured hollow concrete-filled steel tubular columns. J. Civ. Eng. Manag..

[6] Kliukas R., Jaras A., Lukoševičienė O. (2020). Reinforced spun concrete poles—Case study of using chemical admixtures. Materials.

[7] Adesiyun A., Kamiński M., Kubiak J., Łodo A. Laboratory test on the properties of spun concrete. Proceedings of the Third Interuniversity Research Conference.

[8] Adesiyun A., Kamiński M., Kubiak J., Łodo A. (1996). Investigation of Spun-Cast Concrete Structure, School of Young Research Methodology of Concrete Structures.

[9] Kamiński M., Kubiak J., Łodo A., Adesiyun A., Kupski J., Michałek J., Oleszkiewicz T. (1996). Tests of Structural Elements with an Annular Cross-Section Made of Spun Concrete.

[10] Völgyi I., Farkas G., Nehme S.G. (2010). Concrete strength tendency in the wall of cylindrical spun-cast concrete elements. Period. Polytech. Civ. Eng..

[11] Völgyi I., Farkas G. (2011). Rebound testing of cylindrical spun-cast concrete elements. Period. Polytech. Civ. Eng..

[12] Michałek J., Pachnicz M., Sobótka M. (2019). Application of nanoindentation and 2D and 3D imaging to characterize selected features of the internal microstructure of spun concrete. Materials.

[13] Kubiak J., Łodo A., Michałek J. (2015). Production of power spun concrete poles in longitudinally non-openable forms. Build. Mater..

[14] PN-EN 206+A1:2016-12 (2017). Concrete. Specification, Performance, Production and Conformity.

[15] Jarmontowicz A., Krzywobłocka-Laurow R. (1986). Instruction No. 277 Determining the Composition of Hardened Concrete.

[16] (2019). Standard Test Method for Portland-Cement Content of Hardened Hydraulic-Cement Concretete.

[17] (2015). BS 1881-124: 2015 Testing Concrete. Methods for Analysis of Hardened Concrete.

[18] (2019). PN-EN 12390-7: 2019-08 Testing Hardened Concrete. Part 7: Density of Hardened Concrete.

[19] (2010). PN-EN 1936:2010 Natural Stone Test Methods. Determination of Real Density and Apparent Density, and of Total and Open Porosity.

[20] Kozłowski S., Parachoniak W. (1960). Products of basalt weathering in the region of Luban in Lower Silesia. Acta Geol. Pol..

[21] Torquato S. (2002). Random Heterogeneous Materials. Microstructure and Macroscopic Properties.

[22] Łydżba D. (2002). Applications of asymptotic homogenization method in soil and rock mechanics. Scientific Papers of the Institute of Geotechnics and Hydrotechnics.

[23] Różański A. (2010). Random Composites: Representativity, Minimum RVE Size, Effective Transport Properties. Ph.D. Thesis.

[24] Hildebrand T., Rüegsegger P. (1997). A new method for the model-independent assessment of thickness in three-dimensional images. J. Microsc..

[25] Feldkamp L.A., Davis L.C., Kress J.W. (1984). Practical cone-beam algorithm. J. Opt. Soc. Am. A.

[26] Jamroży Z. (2015). Concrete and its Technology.

[27] Bednarek Z., Krzywobłocka-Laurów R., Drzmała T. (2009). Effect of high temperature on the structure, phase composition and strength of concrete. Zesz. Nauk. Sgsp/Szkoła Główna Służby Pożarniczej.

[28] Bultreys T., Van Hoorebeke L., Cnudde V. (2015). Multi-scale, micro-computed tomography-based pore network models to simulate drainage in heterogeneous rocks. Adv. Water Resour..

[29] Cid H.E., Carrasco-Núñez G., Manea V.C. (2017). Improved method for effective rock microporosity estimation using X-ray microtomography. Micron.

[30] Bossa N., Chaurand P., Vicente J., Borschneck D., Levard C., Aguerre-Chariol O., Rose J. (2015). Micro-and nano-X-ray computed-tomography: A step forward in the characterization of the pore network of a leached cement paste. Cem. Concr. Res..

[31] Neville A.M. (2000). Properties of Concrete.

[32] Łydżba D., Różański A. (2014). Microstructure measures and the minimum size of a representative volume element: 2D numerical study. Acta Geophys..

[33] Łydżba D., Różański A., Stefaniuk D. (2018). Equivalent microstructure problem: Mathematical formulation and numerical solution. Int. J. Eng. Sci..

[34] Różański A., Stefaniuk D. (2016). Prediction of soil solid thermal conductivity from soil separates and organic matter content: Computational micromechanics approach. Eur. J. Soil Sci..

